# Transformer-Based Air-to-Ground mmWave Channel Characteristics Prediction for 6G UAV Communications

**DOI:** 10.3390/s25123731

**Published:** 2025-06-14

**Authors:** Borui Huang, Zhichao Xin, Fan Yang, Yuyang Zhang, Yu Liu, Jie Huang, Ji Bian

**Affiliations:** 1 School of Integrated Circuits, Shandong University, Jinan 250101, China; 202200400073@mail.sdu.edu.cn (B.H.); xinzhichao@mail.sdu.edu.cn (Z.X.); 202432387@mail.sdu.edu.cn (F.Y.); 202200400179@mail.sdu.edu.cn (Y.Z.); 2 National Mobile Communications Research Laboratory, School of Information Science and Engineering, Southeast University, Nanjing 211189, China; j_huang@seu.edu.cn; 3 Purple Mountain Laboratories, Nanjing 211111, China; 4 School of Information Science and Engineering, Shandong Normal University, Jinan 250399, China; jibian@sdnu.edu.cn

**Keywords:** channel characteristic prediction, unmanned aerial vehicle (UAV) air-to-ground channels, millimeter-wave (mmWave), multiple-input multiple-output (MIMO)

## Abstract

With the increasing development of 6th-generation (6G) air-to-ground (A2G) communications, the combination of millimeter-wave (mmWave) and multiple-input multiple-output (MIMO) technologies can offer unprecedented bandwidth and capacity for unmanned aerial vehicle (UAV) communications. The introduction of new technologies will also make the UAV channel characteristics more complex and variable, posing higher requirements for UAV channel modeling. This paper presents a novel predictive channel modeling method based on Transformer architecture by integrating data-driven approaches with UAV air-to-ground channel modeling. By introducing the mmWave and MIMO into UAV communications, the channel data of UAVs at various flight altitudes is first collected. Based on the Transformer network, the typical UAV channel characteristics, such as received power, delay spread, and angular spread, are then predicted and analyzed. The results indicate that the proposed predictive method exhibits excellent performance in prediction accuracy and stability, effectively addressing the complexity and variability of channel characteristics caused by mmWave bands and MIMO technology. This method not only provides strong support for the design and optimization of future 6G UAV communication systems but also lays a solid communication foundation for the widespread application of UAVs in intelligent transportation, logistics, and other fields in the future.

## 1. Introduction

6G communication systems aim to establish space–air–ground–sea-integrated networks by integrating cutting-edge technologies, such as full-spectrum resources, ultra-massive antennas, and intelligent reflecting surfaces (IRS). It ultimately achieves Tbps transmission rates, microsecond-level delay, and global deep coverage [1]. As a key enabling scenario of 6G communication, UAVs are attracting extensive attention. Benefiting from its high mobility, flexible deployment, and broad coverage, UAV communication is widely applied in typical scenarios such as target detection, emergency communication, and urban transportation. Moreover, the mmWave band has ultra-wide spectrum resources, which can support low delay and high data transmission rates. It is a promising technology that can overcome the scarcity of spectrum in UAV communication and networks. Therefore, the deep integration of mmWave technology with UAV communication is of the utmost importance [2,3]. Due to the combination of UAV and 6G communications, UAV communication exhibits massive, diverse, and real-time big data features in multiple dimensions such as the space domain, time domain, and frequency domain. To describe the uniqueness of these channels, accurate and efficient modeling and characteristics analysis of UAV air-to-ground channels are essential. As a digital representation of the propagation environment, channel data contains key characteristics such as path loss (PL), delay spread (DS), and channel capacity, which directly determine the quality of service and reliability of the communication links. Hence, developing a high-precision channel model which can mimic these characteristics effectively provides an important guide for parameter optimization and the modulation strategy selection of future 6G UAV communications [4,5].

Existing research teams have carried out a series of studies for UAV communications based on traditional non-predictive channel modeling methods, which can be primarily classified into deterministic channel models and stochastic channel models. Deterministic channel models are generally realized based on stored channel measurements and ray-tracing simulations [6]. In Ref. [7], UAV A2G channel measurements at 26 GHz with a bandwidth of 1 GHz was carried out. The multipath propagation characteristics, path loss, and channel sparsity were analyzed. Channel measurements in an anechoic chamber using a robotic arm to simulate the real movements of UAVs were carried out in [8]. The measurements campaign was performed at 28 GHz with a bandwidth of 300 MHz and focused on investigating the PL and Doppler spread at various distances. Channel simulations for low altitude UAVs in a suburban environment were conducted using the ray-tracing method in [9]. It focused on examining the power delay profile (PDP) and root mean square (RMS) DS. Additionally, in stochastic channel modeling, geometry-based stochastic model (GBSM) has been widely applied. In Ref. [10], a novel non-stationary multi-mobility UAV-to-ship GBSM channel model was proposed. Based on this model, typical wireless channel characteristics were analyzed. In Ref. [11], a non-stationary multi-UAV cooperative channel model for 6G massive MIMO mmWave communications was proposed. The results indicated that the model effectively captured 3D continuously arbitrary trajectories and self-rotation characteristics of multi-UAVs, validated by close agreements between simulation results and ray-tracing-based data. In Ref. [12], a geometry-based non-stationary channel model for 3D UAV mmWave communications was proposed. Iterative calculation methods for time-varying geometric parameters (communication distances, propagation angles) and channel parameters (path delays, powers) were derived. The results showed that the reconstructed channel is consistent with the theoretical one. In summary, traditional channel models have notable limitations. For deterministic models, channel measurements consume significant human, material, and financial resources, while channel simulations such as ray-tracing algorithms are computationally intensive and time-consuming. For stochastic models, GBSMs are widely used but rely heavily on specific scenario parameters in unknown frequency bands and UAV environments. Due to the 3D aeronautic trajectory, multi-mobility of Rx/Tx, and complex scattering environment of UAV communications, the UAV channel modeling especially in the mmWave band still faces numerous challenges.

To overcome the limitations of traditional channel modeling methods, predictive channel modeling methods based on deep learning (DL) are gaining increasing attention. DL methods can automatically establish nonlinear mapping relationships between channel characteristics and complex environments, reducing reliance on manually parameterized models and specific scenarios. Additionally, the DL-based predictive channel model can capture the dynamic variation of the UAV channel and meet real-time requirements [13,14,15,16]. In Refs. [17,18], the generative adversarial network (GAN) was introduced into wireless channel modeling. The key channel parameters such as delays, gains, and angles were generated through adversarial training. The ChannelGAN model was proposed in [19], which incorporated the Earth-Mover distance loss function into the training process to enhance the model stability. In Ref. [20], a machine learning-based prediction method for PL and DS in A2G mmWave channels was proposed, while a machine learning-based PL prediction model for urban street canyon at 28 GHz was proposed in [21]. The results indicated that the proposed architectures could effectively capture complex propagation characteristics. Meanwhile, in Ref. [22], the artificial neural network (ANN) was applied to predict the PL at 881.52 MHz in a rural environment. In Ref. [23], a long short-term memory (LSTM) model was proposed to predict channel characteristics at 28 GHz, including PL, DS, angle spread (AS), and Rician K-factor (KF). This study indicated that the LSTM model can obtain better performance than both convolutional neural network (CNN) and radial basis function (RBF) models in terms of prediction accuracy, particularly for non-stationary urban propagation scenarios. Similarly, the LSTM model was used to achieve high-precision predictions of channel characteristics in [24,25]. In Ref. [26], an AI-enabled data-driven modeling framework was proposed. The channel characteristics (PL, RMS DS, and angles) were predicted. Meanwhile, a convolutional neural network–recurrent neural network (CNN-RNN) model was used for CSI prediction in the same mMIMO channels at 2.35 GHz in [27]. In summary, the current DL-based channel modeling research focuses on sub-6 GHz and single-frequency-band scenarios, lacking comprehensive investigations on mmWave bands and multi-frequency band fusion data conditions. Meanwhile, Transformer-based predictive modeling which can provide the self-attention mechanism for mmWave and multi-band integrated channel modeling remains lacking.

Due to the significant 3D non-stationary characteristics of communication links between UAVs and vehicles, multipath components (MPCs) exhibit dynamic birth and death processes caused by the high mobility of Tx/Rx. In addition, the high PL in mmWave band further enhances signal fluctuations [28]. The fusion data across multiple mmWave bands lead to larger fluctuations in channel characteristics. This brings new challenges for neural network design, demanding robust nonlinear mapping capabilities to address complex input–output relationships [29,30]. Transformer-based networks can provide a feasible solution for addressing this gap. The self-attention mechanism in Transformer globally models relationships between elements in input sequences. It dynamically assigns weights to enable DL models to focus on specific parts of input data that are more relevant to the task at hand [31]. This enhances prediction accuracy for mmWave channel dynamic characteristics, such as RMS DS and RMS AS. In this paper, typical channel characteristics across 10 GHz, 28 GHz, and 38 GHz are predicted. A detailed analysis of model prediction accuracy is conducted, respectively. Typical channel characteristics in mmWave bands are thoroughly analyzed. The Transformer-based model is proposed to predict fused data across three frequency bands. The proposed model based on Transformer is compared with BP-NN, CNN and Attention-BiLSTM models. It significantly improves prediction accuracy and adaptability in complex scattering environments. This is due to its self-attention capacity. The self-attention mechanism enables the model to better capture complex feature relationships, resulting in superior performance in challenging scenarios. The main contributions in this paper are listed as follows:

1. The channel data obtained from Wireless InSite (WI) simulation are verified by the corresponding UAV channel measurements. Based on the validated dataset, extensive channel data in mmWave frequency bands and MIMO case are further achieved. The channel propagation characteristics of different frequency bands (10 GHz, 28 GHz, and 38 GHz) are analyzed respectively.
2. A Transformer-based neural network architecture for 6G mmWave UAV channel characteristics prediction is proposed. Transmitter and receiver (Tx-Rx) coordinates, LoS distance, Tx-Rx altitude difference, location tag, and frequency band are used as the network’s input. Received power, RMS DS, and RMS AS are defined as the output. The model captures long-range dependencies via its self-attention capacity, overcoming limitations of traditional geometry-based modeling in handing non-stationarity and nonlinear mappings. Eventually, UAV channel characteristics can be systematically analyzed in campus-wide area.
3. A multi-dimensional comparative analysis of mmWave UAV channel characteristics is further conducted. It focuses on the impacts of different parameters such as the UAV flight position, flight altitude, and mmWave frequency bands. Meanwhile, based on the simulated data, the prediction accuracy of the Transformer-based predictive model is validated quantitatively.
4. The prediction performance of the Transformer-based predictive model is comprehensively compared with BP-NN, CNN, and Attention-BiLSTM models using the fusion channel datasets. The Transformer-based prediction capability is validated through this experiment. Furthermore, the comparisons not only highlight the advantages of the proposed model but also validate its feasibility and effectiveness in actual applications.

The rest of this paper is organized as follows. Section 2 introduces the reconstruction of the campus scenario using the ray-tracing method and the mmWave UAV A2G channel datasets construction. Typical mmWave UAV channel characteristics are analyzed and the Transformer-based predictive channel model are proposed in Section 3. Section 4 analyzes and evaluates the mmWave UAV channel characteristics and the prediction performance of the proposed model. Finally, conclusions are drawn in Section 5.

## 2. mmWave UAV Communications Architecture and Channel Datasets Construction

### 2.1. Descriptions of mmWave UAV Communications Architecture

**Figure 1:**
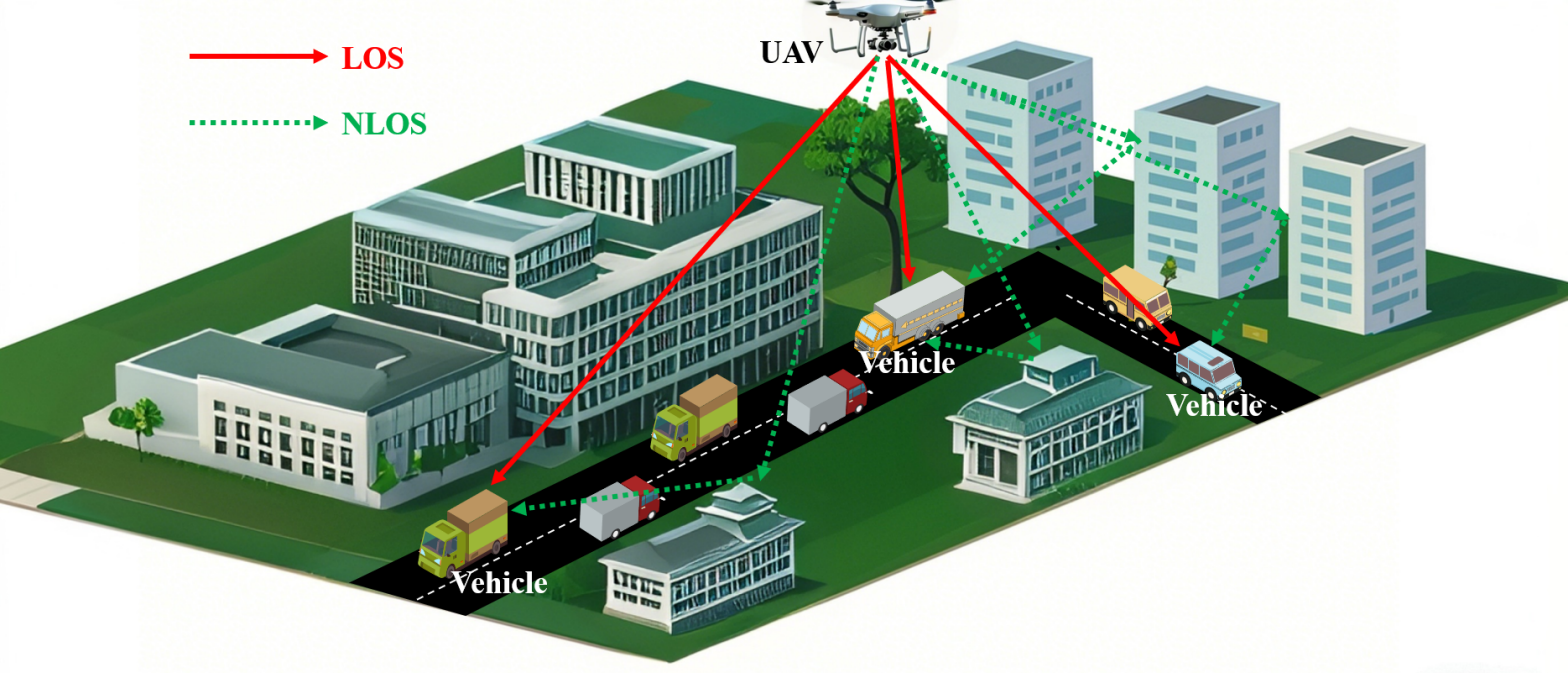
Architecture of mmWave UAV A2G communications network.

UAV communication is deployed in the campus scenario, where the UAV is used to hover in the air to simulate an aerial base station for channel transmission with the ground mobile terminal. The architecture of mmWave UAV A2G communications in the campus scenario is shown in Figure 1. The UAV side is set as Tx using the mmWave frequency, and the vehicle side is set as Rx. Affected by complex factors such as buildings, trees, and others in the campus scenario, the communication link contains two paths: The line-of-sight (LoS) path represents an unblocked communication link between the UAV and the vehicle. It has the strongest signal power, which ensures high quality of communications. The non-line-of-sight (NLoS) path indicates that the signal is affected by buildings, trees, etc. It is transmitted indirectly through reflection, diffraction, etc. This path contains single-reflection paths and multi-reflection paths. Because of the high-PL characteristic of the mmWave band, the multi-reflection paths have large PL. The signal power reaching the ground vehicle is extremely weak, and the quality of the communications is low.

### 2.2. Ray Tracing-Based mmWave UAV Channel Data Acquisition

#### 2.2.1. Measurement Validation for Ray Tracing Simulation

Wireless Insite (WI) is a high-performance radio frequency simulation platform developed by REMCOM. It is specifically built for channel modeling and propagation characteristic analysis of wireless communication systems, and is widely used in channel modeling research [32,33,34]. Based on the ray tracing model, the software has functions such as environmental modeling and rich parameter settings for antenna and carrier models. It can output results such as received power and delay for each received path. It is widely used in simulating indoor and outdoor wireless communication scenarios, supports technologies such as 5G and MIMO, and is a core tool for analyzing electromagnetic wave propagation and communication system performance.

**Figure 2:**
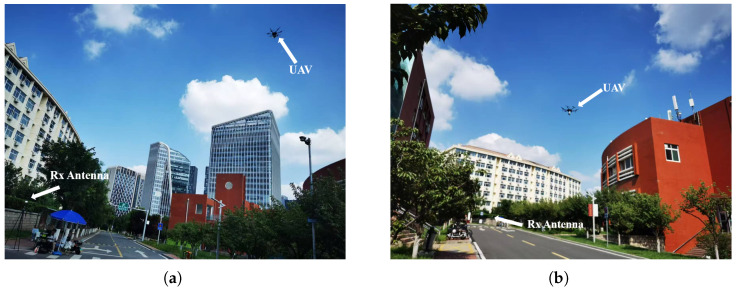
Illustration of UAV air-to-ground channel measurements. (**a**) Measurement scenario 1. (**b**) Measurement scenario 2.

**Figure 3:**
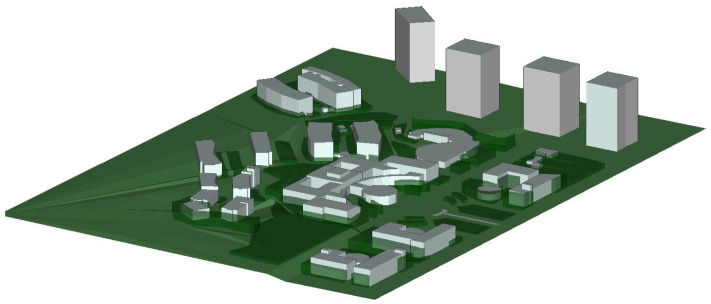
Three-dimensional simulation environment for the campus scenario.

**Figure 4:**
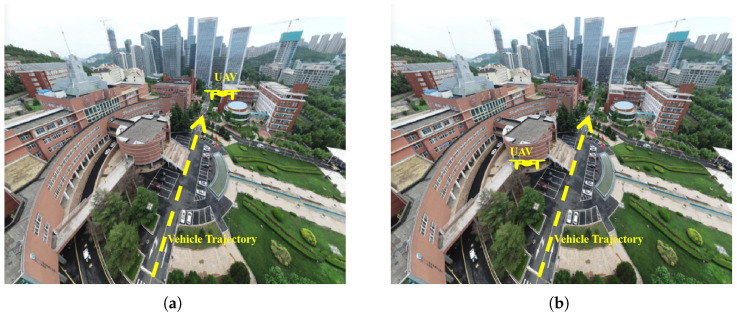
The image of actual campus scenario acquired by UAV. (**a**) Relative position of UAV and vehicle at flight position 1. (**b**) Relative position of UAV and vehicle at flight position 2.

**Figure 5:**
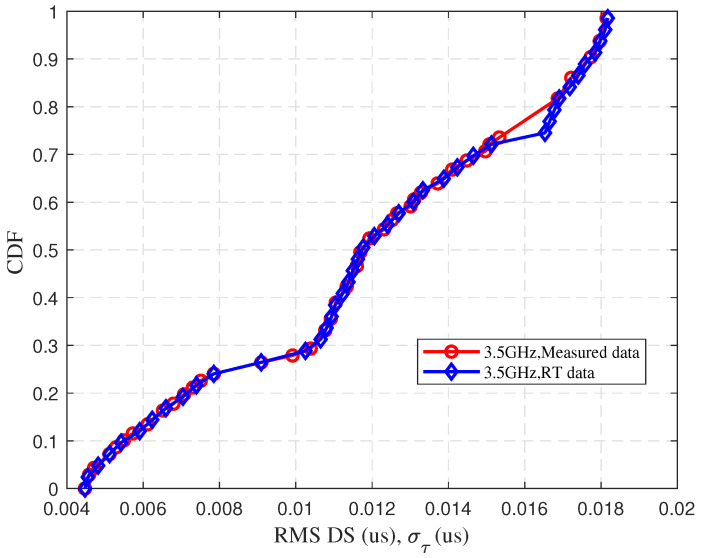
Comparison of measured data and RT data for RMS DS at 3.5 GHz.

To validate the accuracy of the channel data obtained by the WI, UAV A2G communication channel measurements are performed in the campus scenario. The measurement location is selected on the main road of Shandong University Software Campus, which is safe and representative. The scenario includes dense buildings, moving vehicles, trees and other interfering factors. The UAV A2G communication channel measurement scenario is shown in Figure 2. During the measurement, the UAV is used as a Tx, the frequency band is set to 3.5 GHz with a bandwidth of 100 MHz, the transmit power is set to 30 dBm, and it flies horizontally at a height of 20 m with a speed of 5 m/s. The Rx is 1.5 m above the ground, and both the Tx and Rx are equipped with omnidirectional antennas. In addition, based on the ray-tracing (RT) software, the scenario reconstruction of the campus of Shandong University Software Campus is carried out. The reconstructed scenario is shown in Figure 3. The detailed simulation parameter settings are given in Table 1. The actual campus scenario map of the UAV aerial photography is given in Figure 4, and the relative position between the UAV position and the vehicle trajectory during the simulation is given. The measured data and RT data are compared as shown in Figure 5. The results show that the good fit between the measured and RT data proves the usability of the WI and the usefulness of the RT data. The Shandong University Software Campus scenario reconstructed in the simulator is also verified. It indicates that the reconstructed campus scenario in WI software can effectively simulate real environments. Additionally, the WI simulator also shows high accuracy in channel simulation at mmWave frequencies because of its ray-tracing capacity. This has been validated by numerous channel measurement experiments [35,36,37]. Therefore, the WI used to obtain mmWave UAV channel datasets in the campus scenario is highly reliable.

#### 2.2.2. mmWave Channel Datasets Construction

Based on the validated WI 3.4.4 software [38], it is extended to the mmWave band to further acquire channel data in the 6G mmWave band, and the simulation details and related parameter selection are shown in Table 2. The same simulation method is adopted for two UAV flight positions (position 1 and position 2), three frequency bands (10 GHz, 28 GHz, and 38 GHz), and three altitudes (40 m, 70 m, and 100 m) in total of 18 simulation groups. Specifically, the positions are shown in Figure 4. The scenario with the UAV at flight position 1, operating frequency at 28 GHz, and an altitude of 40 m is chosen as an example to introduce the mmWave channel datasets construction process. First of all, the simulation scenario is selected in the software campus of Shandong University. The scenario is reconstructed as shown in Figure 3, and the relative position of the UAV and the vehicle is shown in Figure 4a. The UAV as the Tx flies at an altitude of 40 m, hovering directly in front of the vehicle trajectory. Meanwhile, the vehicle as the Rx travels in a straight line at a speed of 5 m/s. In addition, the frequency is set to 28 GHz, the bandwidth is set to 500 MHz, and the transmit power is set to 10 dBm. For both Tx and Rx, 2 × 2 MIMO antenna arrays spaced at half wavelength are used. Since there are 4 antennas at both the Tx and Rx, the total number of communication channels is 16. The Rx traveling distance is set to 199 m, and the received data is acquired every 1 m, resulting in 200 receiving points. Therefore, each simulation group generates a total of 16 × 200 sets of channel data. $\left(T_{x},T_{y},T_{z}\right)$ and $\left(R_{x},R_{y},R_{z}\right)$ denote the 3D coordinates of Tx and Rx. The distance between Tx and Rx is calculated and is denoted as $X_{LoS}$. The altitude difference between Tx and Rx is denoted as $X_{h}$, the frequency is denoted as $X_{freq}$, and the UAV position tag is denoted as $X_{tag}$. Thus a total of 10 dimensions of data are obtained as input to the network: $\left(T_{x},T_{y},T_{z},R_{x},R_{y},R_{z},X_{LoS},X_{h},X_{freq},X_{tag}\right)$. Finally, 3200 × 2 × 3 × 3 groups of channel data are obtained to construct the mmWave channel datasets for the network training and testing.

In each of these combinations, the datasets are divided into training datasets and testing datasets according to a ratio of 9:1. A total of 2880 datasets from 180 different Rx locations are selected for network training, while the remaining 320 datasets from 10 different Rx locations are used for network testing. In order to reduce the randomness between neighboring data, the training datasets and testing datasets are randomly ordered and then input into the network. The testing datasets output the corresponding predicted channel characteristic values, which are compared with the channel characteristic values obtained from the simulation. After completing the tests for each of the 18 combinations, in each combination, a point is selected every other Rx position. A total of 1600 sets of data are obtained from 50 different Rx positions in each combination. All 18 combinations are combined, resulting in 1600 × 18 sets of data, which are fused to construct a merged datasets. The training datasets and testing datasets in the merged datasets are generated using the same method as described earlier. The model is trained using the same method as described earlier.

The simulation data is generated from typical UAV application scenarios. One scenario is UAV hovering as an aerial base station to communicate with ground mobile vehicles. Three typical mmWave frequency bands, three flight altitudes, and two flight positions are selected. Together, a total of 18 simulation scenarios are created. The resulting dataset effectively maps the campus environment. The relevant works can be extended to single-end UAV mobile communication scenarios. They can also be extended to dual-end mobile scenarios between UAVs and ground vehicles.

## 3. Transformer-Based Prediction of mmWave UAV A2G Channel Characteristics

### 3.1. Descriptions of Typical Channel Characteristics

#### 3.1.1. Received Power

For UAV communications, the signals emitted by the Tx will experience MPCs to reach the Rx after different propagation mechanisms from the channel. The RT method can be utilized to track the received power of each individual path. By summing the received power of all paths, the total power at each Rx location is obtained. This received power directly reflects the channel quality and is employed to evaluate the reliability of the communication link. The total received power is described as

(1) $$P=10\log_{10}\left(\sum_{l=1}^{L}P_{l}\right),$$

where $P_{l}$ denotes the received power of the *l*-th MPCs, and *L* is the total number of MPCs.

#### 3.1.2. Root Mean Squared Delay Spread

In wireless channels, the signal undergoes complex propagation processes like reflection, diffraction, and transmission. These processes form MPCs with different time delays arriving at the Rx location. Due to the varying propagation distances and numbers of reflections in each path, the received signal exhibits a time dispersion phenomenon. This phenomenon causes the inter-symbol interference (ISI) problem. As a core parameter to quantify the time dispersion, RMS DS is the core index for evaluating small-scale fading. It is directly related to the channel coherence bandwidth, and the formula is given by

(2) $$\sigma_{RMS\;DS}=\sqrt{\frac{\sum_{l=1}^{L}P_{l}\tau_{l}^{2}}{\sum_{l=1}^{L}P_{l}}-{\left(\frac{\sum_{l=1}^{L}P_{l}\tau_{l}}{\sum_{l=1}^{L}P_{l}}\right)}^{2}},$$

where $\tau_{l}$ denotes the delay of the *l*-th MPCs.

#### 3.1.3. Rician K-Factor

In UAV communications, the Rician K-factor (KF) is a core parameter describing the ratio of the LoS path power and NLoS paths power [39]. It directly reflects the strength of the LoS paths during channel transmission and is expressed as

(3) $$\delta_{KF}=\frac{P_{LoS}}{\sum_{l=1}^{L}P_{l}-P_{LoS}},$$

where $P_{LoS}$ denotes the power value of the LoS path.

#### 3.1.4. Root Mean Squared Angle Spread

In scattering environments, signals undergo multipath propagation, and the azimuth and elevation angles of arrival at the receiver antenna array exhibit discrete distributions. As a second-order statistical measure, the RMS AS quantifies this discreteness using power-weighted angular standard deviations. Angles at both the Tx and Rx are divided into azimuth and elevation angles. The formula is given by

(4) $$\sigma_{RMS\;ASA}=\sqrt{\frac{\sum_{l=1}^{L}P_{l}\theta_{l}^{2}}{\sum_{l=1}^{L}P_{l}}-{\left(\frac{\sum_{l=1}^{L}P_{l}\theta_{l}}{\sum_{l=1}^{L}P_{l}}\right)}^{2}},$$

(5) $$\sigma_{RMS\;ASE}=\sqrt{\frac{\sum_{l=1}^{L}P_{l}\varphi_{l}^{2}}{\sum_{l=1}^{L}P_{l}}-{\left(\frac{\sum_{l=1}^{L}P_{l}\varphi_{l}}{\sum_{l=1}^{L}P_{l}}\right)}^{2}},$$

where $\theta_{l}$ denotes the *l*-th angle spread of azimuth (ASA), and $\varphi_{l}$ denotes the *l*-th angle spread of elevation (ASE). Respectively, the root mean squared azimuth angle of arrival spread (RMS AAOA), the root mean squared elevation angle of arrival spread (RMS EAOA), the root mean squared azimuth angle of departure spread (RMS AAOD), and the root mean squared elevation angle of departure spread(RMS EAOD) can be acquired.

The above typical channel characteristics can be represented as seven dimensions of data as network outputs: $\left(P,\sigma_{RMS\;DS},\delta_{KF},\sigma_{RMS\;AAOA},\sigma_{RMS\;EAOA},\sigma_{RMS\;AAOD},\sigma_{RMS\;EAOD}\right)$.

### 3.2. Model Design

**Figure 6:**
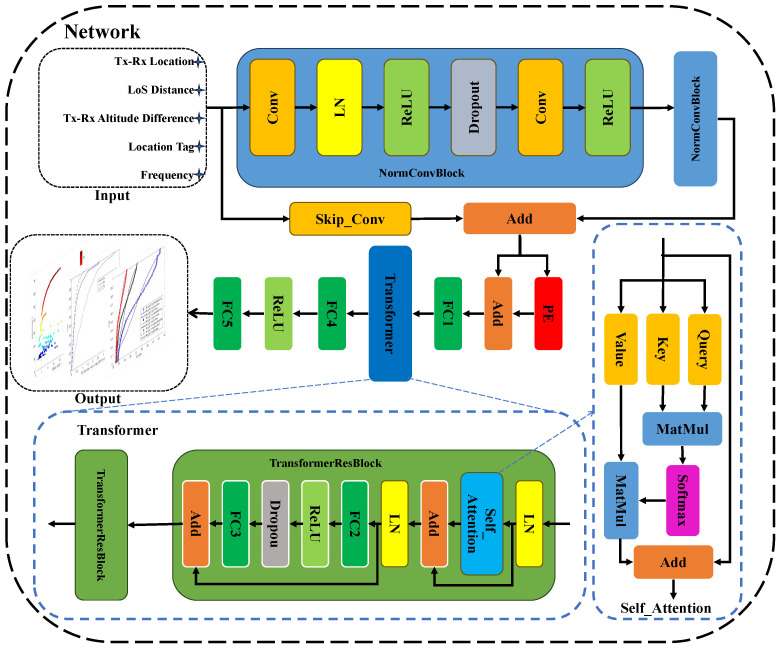
The network structure of the channel prediction.

Based on the constructed 6G mmWave UAV communication channel datasets, a Transformer-based channel characteristics prediction model is proposed for the first time. The structure of this network model is shown in Figure 6. The model combines CNN and Transformer. CNN captures local dependencies in sequence data, while the Transformer extracts global correlations. These capabilities improve the prediction performance of the model. The model can extract environmental features from the Tx and Rx coordinates, LoS distances, Tx and Rx altitude differences, location tags, and frequency bands. Then the received power, RMS DS, KF, and RMS AS in campus scenarios are predicted. Before feeding the dataset into the network, we first perform one-hot encoding on the location tags to project the one-dimensional location tags into two dimensions. In this two-dimensional space, the one-hot vectors of different location tags are orthogonal to each other, resulting in clearer and more separable feature representations. This orthogonality enables neural networks to more easily capture the differences between locations, thereby enhancing the learning efficiency of location-specific information. At this time, the network inputs are projected from 10 dimensions to 11 dimensions. Then, the network inputs are normalized to the [0, 1] interval. This normalization prevents large-scale features from dominating training, thereby improving the model’s accuracy.

The normalized data undergoes feature extraction and preliminary processing through two NormConvBlock modules. Specifically, the NormConvBlock is a customized convolution module. It contains convolution (Conv) layers, normalization (LN) layers, ReLU activation functions, and Dropout modules. The first convolution layer projects the input data from 11 dimensions to 64 dimensions. In subsequent modules within the NormConvBlock, the data remains in 64 dimensions. Each NormConvBlock contains two 4 × 4 convolution layers with 64 channels. The convolution padding is set to “same” to ensure the sequence length stays unchanged after the convolution operation. When the convolution kernel slides over the input sequence, edge elements are convolved fewer times than central elements, which may cause edge information loss. The “same” padding mitigates this issue by filling the sequence ends with zeros. This ensures edge elements are fully convolved, thereby avoiding information loss. The details of the proposed model are in Table 3. The first convolution layer enhances the representation of the model by capturing the local correlations of the input data. The second convolution layer enables further extraction and fusion of data characteristics, abstracting high-level features among data, such as periodic upward or downward trends. The layer normalization stabilizes the distribution of data features and makes the gradient more stable. The ReLU activation function enables to capture the nonlinearity in the data and improves the nonlinear representation of the model. The Dropout module randomly sets neuron outputs to zero with a probability of 0.01. This method effectively prevents the overfitting of the data and enhances the generalization ability and robustness of the model. Subsequently, The Skip-Conv module is used to establish a skip connection with the data processed by the NormConvBlock module. The Skip-Conv module ensures that the data dimensions of the two branches are consistent. Skip connection enables to further alleviate the vanishing gradient problem in the deep network and enhances the network learning capacity. Finally, the NormConvBlock module outputs 64-dimensional data for subsequent processing.

Following the previous processing steps, the data undergoes positional encoding (PE) and an addition (Add) layer before entering the FC layer. Then, the processed data flows into the Transformer module. Since the Transformer itself cannot perceive positional information, the PE module addresses this gap. It allows the model to capture patterns and regularities in the input sequences more accurately. These operations improve the model’s accuracy. After passing through the PE module and the Add layer, the data remains 64-dimensional for subsequent processing. Then an FC layer preceding the Transformer module projects the data from 64 dimensions to 11 dimensions. The Transformer module mainly contains the self-attention module, which projects each element in the input sequence into a query (Q) vector, a key (K) vector, and a value (V) vector, respectively, by three different linear transformations. Then the similarity between each query vector and all key vectors is calculated to obtain the attention score. Next, the $\sqrt{d_{k}}$ is used as the scaling factor to prevent excessively large dot products from causing gradient vanishing or exploding. Finally the scaled attention scores are normalized by the Softmax function [40,41]. The formula is defined as

(6) $$\mathrm{Attention}\left(Q,K,V\right)=\mathrm{softmax}\left(\frac{QK^{T}}{\sqrt{d_{k}}}\right)V,$$

where *Q* denotes the query vector, *K* represents the key vector, and *V* is the value vector. The $\sqrt{d_{k}}$ is the scaling factor to prevent excessively large dot products. The self-attention layer computes six different attention heads, with each head having key and value vectors of 32 dimensions. It is introduced to dynamically assign attention weights so that the model can focus on the interrelationships between elements at different locations in the sequence. It enhances the ability to capture long-range dependencies and improves the model’s prediction accuracy. Additionally, the first FC layer in the Transformer module projects data from 11 dimensions to 33 dimensions, and the second FC layer projects data from 33 dimensions to 11 dimensions. This steps enhance the feature representation of the model, while preventing overfitting of the data and enhancing the generalization ability. The data passes through the Transformer module, enhancing the understanding of the whole sequence, and the data is projected from 64 dimensions to 11 dimensions.

The data is processed by the Transformer module. It then enters a feed-forward neural network. This network includes FC layers and ReLU activation functions. Since self-attention is inherently linear, the feed-forward network introduces nonlinear transformations. This enhances the model’s ability to fit complex data. The first FC layer projects the data from 11 dimensions to 33 dimensions. The second FC layer adjusts dimensions, projecting the data from 64 dimensions to 7 dimensions. Finally, the seven-dimensional data undergoes denormalization to finish predictions.

To facilitate the effective assistance of sensory information in channel characteristic prediction during model training, Mean Squared Error (MSE) is selected as the loss function. The MSE formula is provided as

(7) $$L_{MSE}=\frac{1}{n}\sum_{i=1}^{n}{\left(y_{i}-{\hat{y}}_{i}\right)}^{2},$$

where ${\hat{y}}_{i}$ represents the predicted value of the *i*-th sample, $y_{i}$ represents the simulated value of the *i*-th sample, and *n* denotes the number of channel samples used for testing. A small $L_{MSE}$ value indicates better prediction performance of the network.

## 4. Results and Analysis

### 4.1. Evaluation Metrics

Three evaluation metrics are chosen to comprehensively validate the prediction performance of the proposed model. These metrics assess the model’s prediction performance across different flight positions, flight altitudes, and frequency bands. Additionally, the accuracy of different neural network models are quantitatively compared and evaluated. The three evaluation metrics are RMSE, MAPE and MAE. In the mmWave UAV communication channel prediction, these metrics comprehensively evaluate the accuracy of the proposed model and also objectively compare the prediction performance of different neural network models.

RMSE is a commonly used metric to measure the deviation between the predicted value and the real value. It can directly reflect the average deviation between the predicted value and the real value, and make it easy to understand the magnitude of the error clearly. Furthermore, RMSE amplifies the impact of extreme error through the squared arithmetic. The zero-tolerance characteristic of RMSE for large errors is well-suited to mmWave UAV communication systems. This unique characteristics forces the model to enhance the robustness of the extreme values to ensure the stable output of prediction performance under extreme conditions. The RMSE formula is defined as

(8) $$\mathrm{RMSE}=\sqrt{\frac{1}{n}\sum_{i=1}^{n}{\left({\hat{y}}_{i}-y_{i}\right)}^{2}},$$

where ${\hat{y}}_{i}$ represents the predicted value of the *i*-th sample, $y_{i}$ represents the simulated value of the *i*-th sample, and *n* denotes the number of channel samples used for testing. A smaller RMSE value indicates better prediction performance.

MAPE is a standardized metric of the relative error between the predicted value and the true value. It directly quantifies the degree of prediction deviation through the form of percentage, and its core principle is to take the absolute percentage of each error term and then average it. This property naturally eliminates the influence of the magnitude, enabling more equitable comparisons of prediction error ranges across different channel characteristics. The MAPE formula is defined as

(9) $$\mathrm{MAPE}=\frac{1}{n}\sum_{i=1}^{n}\left|\frac{{\hat{y}}_{i}-y_{i}}{y_{i}}\right|\times 100\%,$$

where ${\hat{y}}_{i}$ represents the predicted value of the *i*-th sample, $y_{i}$ represents the simulated value of the *i*-th sample, and *n* denotes the number of channel samples used for testing. Similarly, a smaller MAPE value indicates better prediction performance.

MAE is the arithmetic mean of absolute deviations between the predicted value and the true value. Specifically, it calculates the average by summing absolute errors. This method outputs a directionless and unweighted metric of average deviation. The MAE formula is defined as

(10) $$\mathrm{MAE}=\frac{1}{n}\sum_{i=1}^{n}\left|{\hat{y}}_{i}-y_{i}\right|,$$

where ${\hat{y}}_{i}$ represents the predicted value of the *i*-th sample, $y_{i}$ represents the simulated value of the *i*-th sample, and *n* denotes the number of channel samples used for testing. Similarly to RMSE and MAPE, a smaller MAE value indicates better prediction performance.

### 4.2. Transformer-Based Channel Prediction Performance and Channel Characteristics Analysis

#### 4.2.1. Different UAV Flight Positions

**Figure 7:**
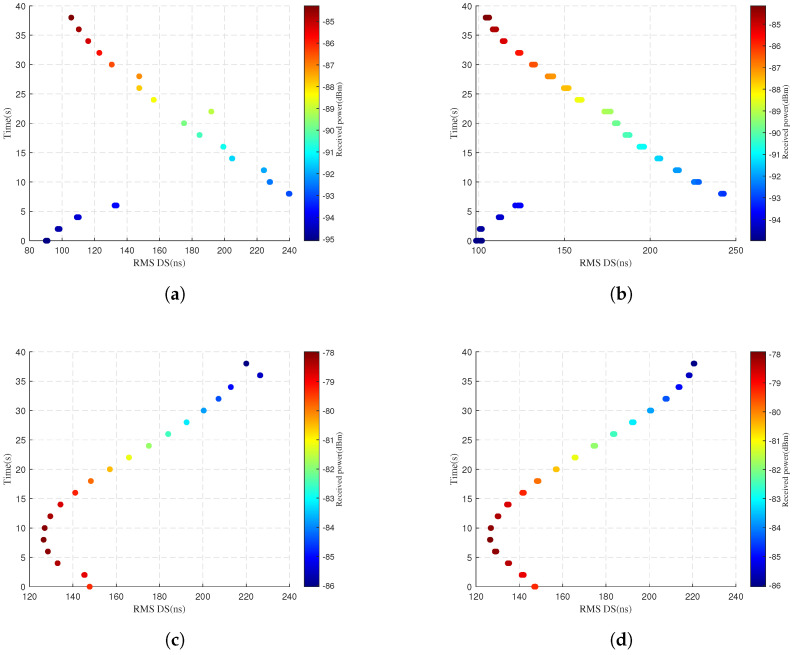
Comparison of RMS DS and received power at different flight positions. Simulation values are displayed in the left column, while predicted values are presented in the right column. (**a**) Actual flight position 1. (**b**) Predicted flight position 1. (**c**) Actual flight position 2. (**d**) Predicted flight position 2.

Figure 7 compares simulated and predicted RMS DS and received power for different UAV flight positions. The left column displays simulated values, while the right column shows predicted values. The horizontal axis represents RMS delay spread, the vertical axis denotes the vehicle travel time, and the color intensity of scatter points indicates the received power levels. Based on the relative positions of the UAV and vehicle during simulation in Figure 4, in Figure 7a at flight position 1, the received power increases progressively as the vehicle approaches the UAV. In Figure 7c at flight position 2, the received power first increases then decreases as the vehicle approaches and departs. These phenomena indicate that mmWave signals follow free-space path loss. The PL increases with an increasing propagation distance. In Figure 7a, RMS DS decreases at distant UAV position 1. Possible reasons include the significant distance from the UAV and fewer scatterers. mmWave signals experience a sharp decrease with an increasing propagation distance. So the MPCs are reduced. A comparison of Figure 7a–d shows that the proposed model is accurate in predicting the RMS DS and received power.

**Figure 8:**
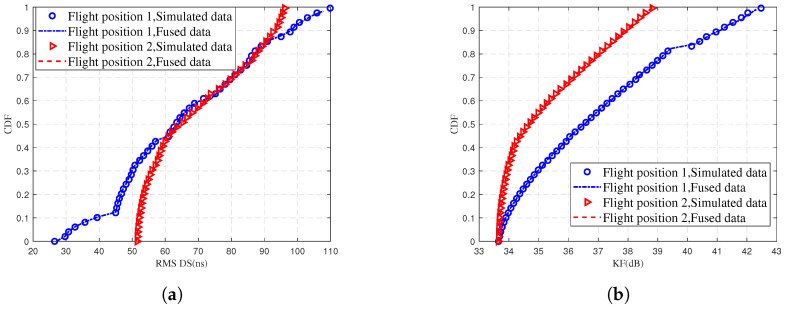
Comparison of simulated data and fused data for (**a**) RMS DS and (**b**) KF at different UAV flight positions.

Figure 8 illustrates the CDF comparison of the RMS DS and KF for different UAV flight position, where the fusion data represents the fusion of predicted data and training data. Figure 8a shows that the RMS DS is concentrated in 30–90 ns for location 1 and 50–90 ns for location 2. In Figure 8b, it is shown that the KF in location 1 is smaller than location 2. The possible reason for this phenomenon is that Location 2 is closer to the school teaching building. The teaching building significantly affects Location 2, leading to richer scattering paths and greater losses. Ultimately, this results in more MPCs. Therefore, the quality of UAV communications is degraded. The fitting of the simulated values and fused data in Figure 8 responds to the fact that the proposed model is accurate in the prediction of the RMS DS and the KF. In order to accurately respond to the model prediction performance for the RMS DS and the KF, Table 4, Table 5, Table 6 and Table 7 present the quantitative results in detail.

Table 4 and Table 5 present the quantitative prediction performance of RMS DS under different flight positions, different communication frequencies, and different flight altitudes. It can be seen that the model’s prediction performance for RMS DS is better at flight position 2 than at flight position 1, and better at 38 GHz than in other frequency bands. The reason may be that the higher the frequency band, the smaller the number of MPCs and the lower the data fluctuation.

Table 6 and Table 7 present the quantitative prediction performance of KF under different flight positions, different communication frequencies, and different flight altitudes. From the table, it can be seen that there are very few values of RMSE and MAE that are greater than 1 dB at flight position 1. The rest of the values are close to zero. This phenomenon indicates that the model has excellent predictive performance for the KF.

#### 4.2.2. Different UAV Flight Altitudes

**Figure 9:**
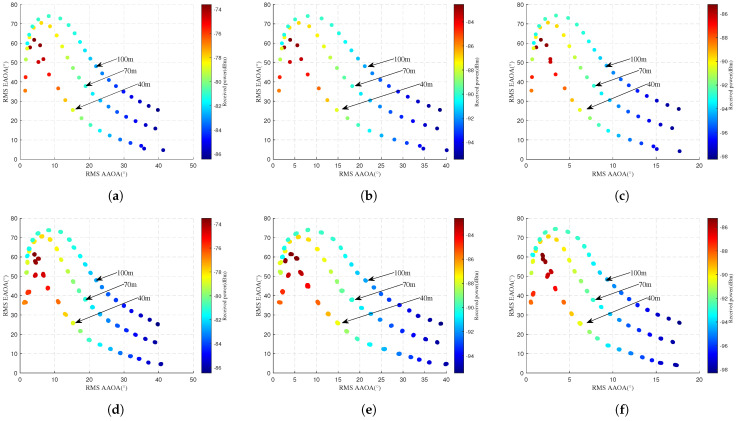
Comparison of RMS AOA and received power at different flight altitudes. Simulated values are displayed in the first row, while predicted values are presented in the second row. (**a**) Actual 10 GHz. (**b**) Actual 28 GHz. (**c**) Actual 38 GHz. (**d**) Predicted 10 GHz. (**e**) Predicted 28 GHz. (**f**) Predicted 38 GHz.

**Figure 10:**
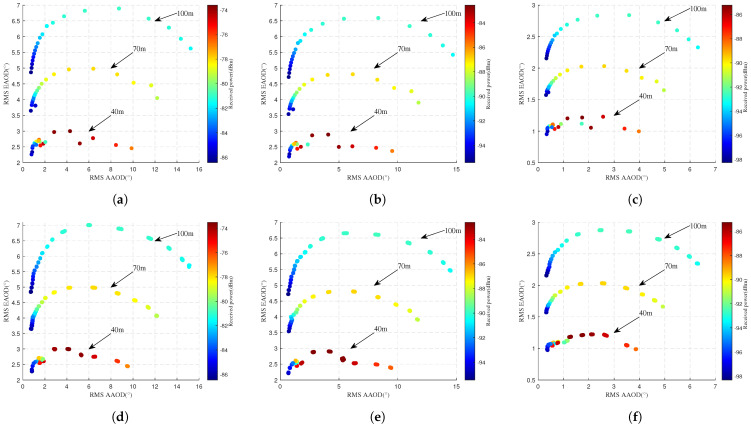
Comparison of RMS AOD and received power at different flight altitudes. Simulated values are displayed in the first row, while predicted values are presented in the second row. (**a**) Actual 10 GHz. (**b**) Actual 28 GHz. (**c**) Actual 38 GHz. (**d**) Predicted 10 GHz. (**e**) Predicted 28 GHz. (**f**) Predicted 38 GHz.

Figure 9 and Figure 10 compare simulated and predicted RMS AS and power for different UAV flight altitudes. The first row displays simulated values, while the second row shows predicted values. The horizontal axis represents RMS ASA, the vertical axis denotes RMS ASE, and the color intensity of scatter points indicates received power levels. In Figure 9a–c and Figure 10a–c, it can be seen that the received power decreases as the UAV flight altitude increases. This further verifies that mmWave signals follow the law of free-space path loss where PL increases with the increasing propagation distance. It is also found that the RMS EAOA, RMS AAOD, and RMS EAOD gradually increase with increasing UAV altitude, and the RMS AAOA remains basically unchanged. This may be because higher UAV positions tend to align with mid-to-upper sections of tall buildings on campus. These locations are more significantly influenced by tall structures, increasing the diversity of scattering paths. Comparison of the simulated and predicted values in Figure 9 and Figure 10 shows that the proposed model is accurate in predicting RMS AS and the received power for different altitudes. In order to accurately respond to the model prediction performance for the RMS AOA and the received power, Table 8, Table 9, Table 10, Table 11, Table 12 and Table 13 present the quantitative results in detail.

Table 8 and Table 9 present the quantitative prediction performance of received power under different flight positions, different communication frequencies, and different flight altitudes. As can be seen from the table, the evaluation metrics are all close to zero, indicating that the model performs well for received power prediction, especially for flight position 2.

Table 10 and Table 11 present the quantitative prediction performance of RMS AAOA under different flight positions, different communication frequencies, and different flight altitudes. It can be seen that the model’s prediction performance for RMS AAOA is better at flight position 2 than at flight position 1. The reason for this may be the high variability in the horizontal environment at flight position 1, which leads to high variability in the RMS AAOA. So the model prediction performance decreases.

Table 12 and Table 13 present the quantitative prediction performance of RMS EAOA under different flight positions, different communication frequencies, and different flight altitudes. It can be seen that the model’s prediction performance for RMS EAOA is good. This indicates that there is little change in the environment in the vertical direction and little change in the data. The model has strong prediction performance.

#### 4.2.3. Different UAV Communication Frequencies

**Figure 11:**
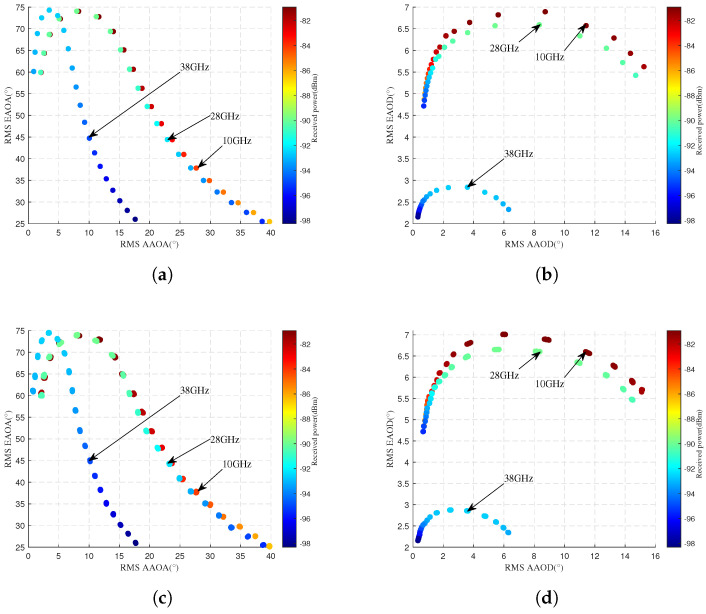
Comparison of angle and received power at different flight altitudes. Simulated values are displayed in the first row, while the predicted values are presented in the second row. (**a**) Actual RMS AOA. (**b**) Actual RMS AOD. (**c**) Predicted RMS AOA. (**d**) Predicted RMS AOD.

Figure 11 compares simulated and predicted RMS AS and power for different frequency bands. The first row displays simulated values, while the second row shows predicted values. The horizontal axis represents RMS ASA, the vertical axis denotes RMS ASE, and the color intensity of scatter points indicates received power levels. Figure 11a,c show that the received power decreases as the frequency increases. This indicates higher PL at elevated mmWave frequencies. Additionally, RMS AAOA, RMS AAOD, and RMS EAOD gradually decline with increasing frequency. This may because higher mmWave frequencies increase PL, reducing the number of effective paths. These trends demonstrate the high loss and limited propagation distance of mmWave frequencies. In order to accurately respond to the model prediction performance for the RMS AOD, Table 14, Table 15, Table 16 and Table 17 present the quantitative results in detail.

Table 14 and Table 15 present the quantitative prediction performance of RMS AAOD under different flight positions, different communication frequencies, and different flight altitudes. From the table it can be seen that there are very few values of RMSE and MAE greater than 1° at flight position 1. The rest of the values are close to zero. This phenomenon indicates that the model has excellent predictive performance for the RMS AAOD.

Table 16 and Table 17 present the quantitative prediction performance of RMS EAOD under different flight positions, different communication frequencies, and different flight altitudes. As can be seen from the table, the model exhibits strong prediction performance for RMS EAOD. And the best prediction performance is in the case of 38 GHz and 70 m.

**Figure 12:**
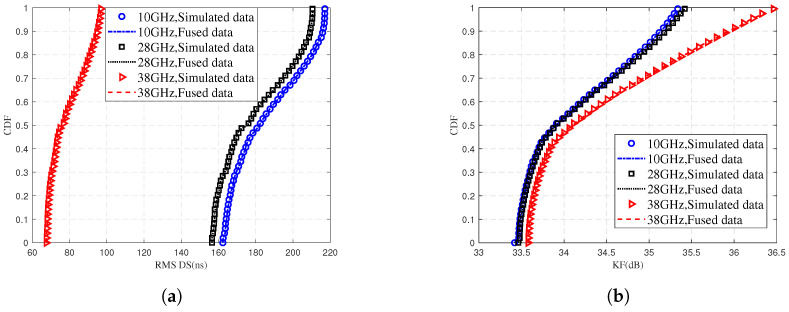
Comparison of simulated data and fused data for (**a**) RMS DS and (**b**) KF at different communication frequencies.

Figure 12 illustrates the CDF comparison of the RMS DS and KF for different frequency bands, where the fusion data represents the fusion of predicted data and training data. It shows that as the frequency increases, curves shift left in Figure 12a and right in Figure 12b. This is due to the increased path loss at higher mmWave frequencies. The NLOS paths attenuate rapidly under this condition. LOS paths become dominant as a result. These combined factors reduce MPCs in high-frequency signals. The fitting of the simulated values and fused data in Figure 12 responds to the fact that the proposed model is accurate in the prediction of the RMS DS and the KF.

### 4.3. Comparison of Channel Prediction Performance

Considering that the proposed model incorporates the architectural features of CNN and Transformer, in order to fully validate the effectiveness and unique advantages of the Transformer-based model, we select a single CNN model, and classical BP-NN and Attention-BiLSTM models for comparison experiments. Evaluation using the merged datasets better demonstrates model robustness. Cumulative probability distribution curves of absolute prediction errors are plotted to reflect the prediction performance of the four models. The absolute error is defined as

(11) $$\mathrm{abs}\left({\mathrm{Error}}_{i}\right)=\left|{\hat{y}}_{i}-y_{i}\right|,$$

where ${\hat{y}}_{i}$ represents the predicted value of the *i*-th sample, and $y_{i}$ represents the simulated value of the *i*-th sample. Smaller horizontal ranges at vertical axis value 1 of the cumulative probability distribution curve indicate better prediction performance. Steeper curve slopes also reflect superior model performance. All three models effectively predict overall trends. However, the proposed model demonstrates significantly better prediction accuracy compared to BP-NN, CNN, and Attention-BiLSTM models.

**Figure 13:**
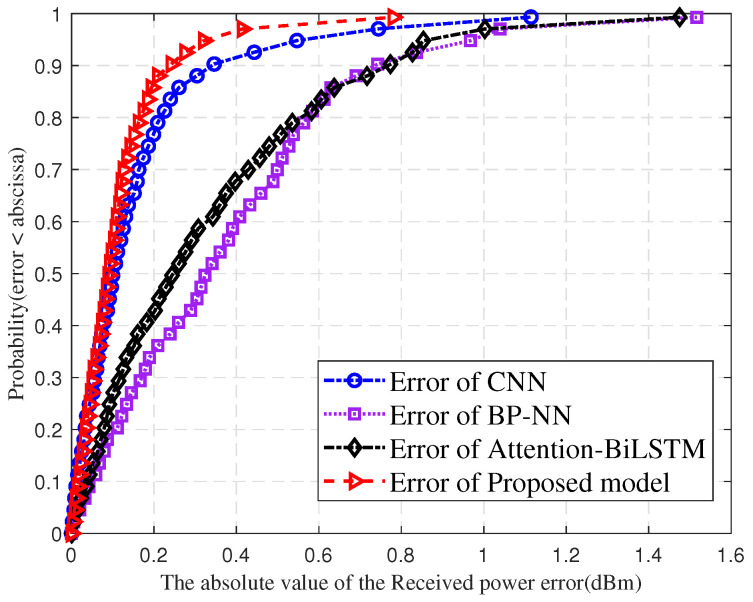
Comparison of errors in received power prediction for different models.

Figure 13 compares cumulative distribution curves of absolute received power prediction errors for BP-NN, CNN, Attention-BiLSTM and the proposed model. The horizontal axis shows absolute error in dBm, while the vertical axis represents cumulative probability. the proposed model’s errors range from 0 to 0.8 dBm. BP-NN errors range from 0 to 1.5 dBm. CNN errors range from 0 to 1.1 dBm and Attention-BiLSTM errors range from 0–1.4 dBm. This demonstrates the proposed model’s prediction performance through tighter low-error concentration and lower large-error tolerance. Results show that BP-NN, CNN, and Attention-BiLSTM exhibit more dispersed error distributions, indicating inferior stability and precision compared to the proposed model.

**Figure 14:**
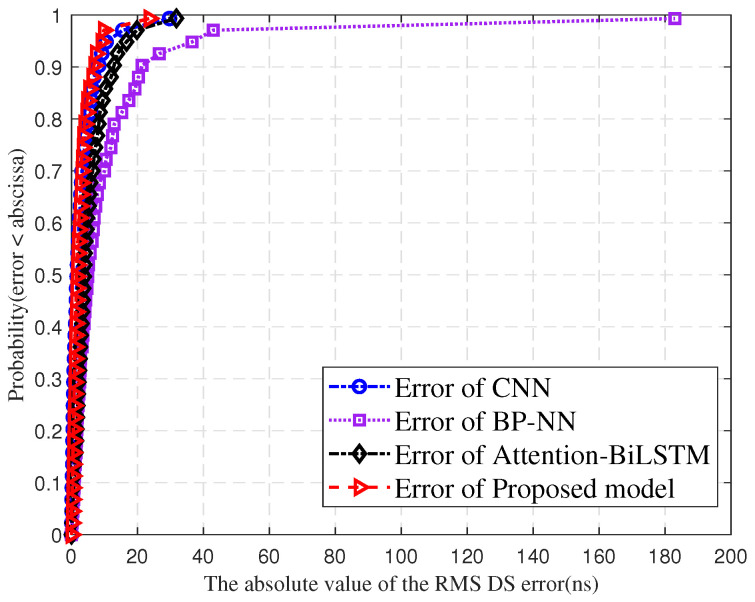
Comparison of errors in RMS DS prediction for different models.

Figure 14 compares the cumulative distribution curves of absolute RMS DS prediction errors for BP-NN, CNN, Attention-BiLSTM, and the proposed model. The horizontal axis shows the absolute error in ns, while the vertical axis represents the cumulative probability. It shows the absolute error curve of the proposed model on the left. BP-NN, CNN, and Attention-BiLSTM error distributions are positioned to the right. The red curve (the proposed model) has a steeper slope than the blue curve (CNN), purple curve (BP-NN), and black curve (Attention-BiLSTM). This indicates better prediction performance of the proposed model compared to other models.

**Figure 15:**
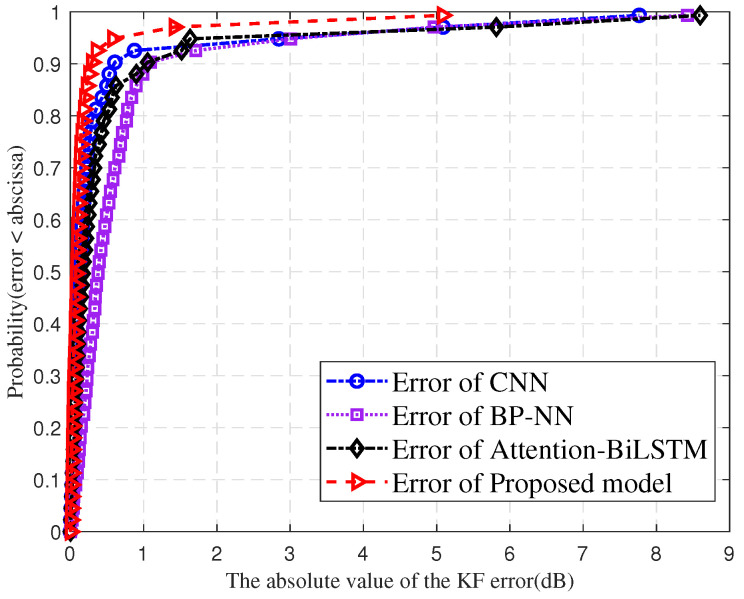
Comparison of errors in KF prediction for different models.

Figure 15 compares the cumulative distribution curves of absolute KF prediction errors for BP-NN, CNN, Attention-BiLSTM, and the proposed model. The horizontal axis shows absolute error in dB, while the vertical axis represents the cumulative probability. The proposed model’s errors range from 0 to 5 dB. BP-NN errors range from 0 to 8.5 dB. CNN errors range from 0 to 7.7 dB, and Attention-BiLSTM errors range from 0 to 8.5 dB. This demonstrates the proposed model’s prediction performance through tighter low-error concentration and lower large-error tolerance. Results show that BP-NN, CNN, and Attention-BiLSTM exhibit more dispersed error distributions, indicating inferior stability and precision compared to the proposed model.

**Figure 16:**
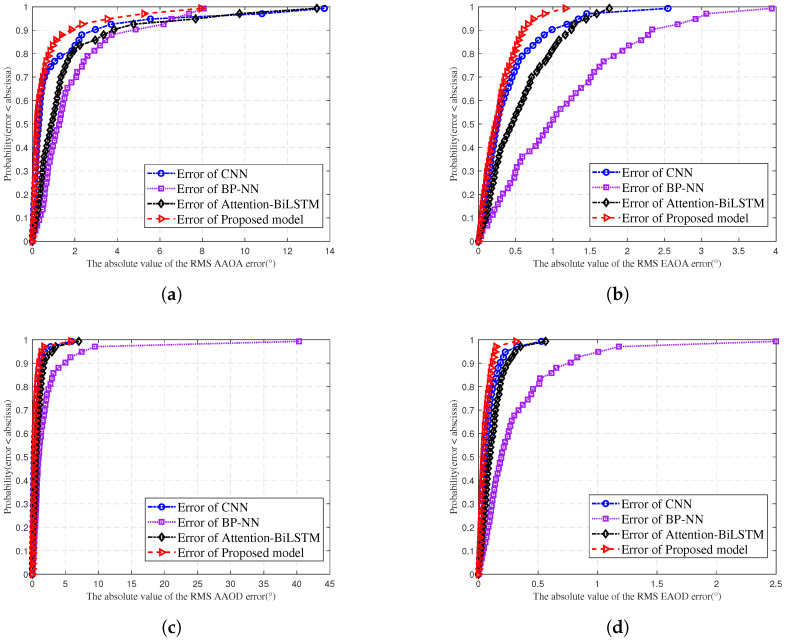
Comparison of errors in (**a**) RMS AAOA prediction, (**b**) RMS EAOA prediction, (**c**) RMS AAOD prediction, and (**d**) RMS EAOD for different models.

Figure 16 compares the cumulative distribution curves of absolute RMS AS prediction errors for BP-NN, CNN, Attention-BiLSTM, and the proposed model. The horizontal axis shows absolute error in ‘°’, while the vertical axis represents the cumulative probability. Figures show the absolute error curve of the proposed model on the left. BP-NN, CNN, and Attention-BiLSTM error distributions are positioned to the right. The red curve (the proposed model) has a steeper slope than the blue curve (CNN), purple curve (BP-NN), and black curve (Attention-BiLSTM). This indicates better prediction performance of the proposed model compared to other models.

To accurately reflect the prediction abilities of the four models for each channel characteristic value, Table 18 shows the details of the quantitative results. It indicates that the proposed model has strong prediction performance for received power, KF, and RMS AS. However, The accuracy of the proposed model for RMS DS is poor. The possible reason is that the RMS DS has an overly large changing trend because of mmWave bands. The evaluation indicators RMSE, MAPE, and MAE are compared, and the accuracy of the proposed model for the seven channel characteristics is significantly better than that of the BP-NN, CNN, and Attention-BiLSTM models.

**Figure 17:**
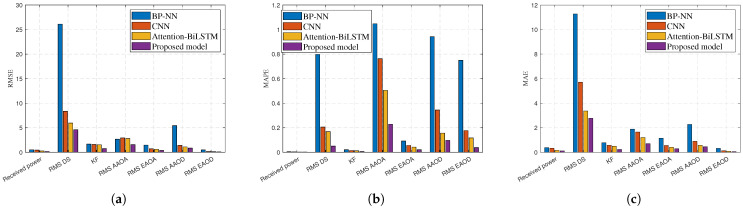
Comparison of (**a**) RMSE, (**b**) MAPE, and (**c**) MAE for BP-NN, CNN, Attention-BiLSTM, and the proposed model using bar charts.

To more intuitively show the magnitude relationships among the evaluation metrics RMSE, MAPE, and MAE, Figure 17 presents the bar charts of RMSE, MAPE, and MAE, respectively. It is clear that the prediction performance of the proposed model for the seven channel characteristics is significantly better than that of the BP-NN, CNN, and Attention-BiLSTM models.

An experiment on the complexity of the proposed model is also conducted. The results show that the core complexity of the proposed model mainly originates from the convolutional layers and self-attention layer. Specifically, when the sequence length is large, the time complexity primarily depends on the self-attention layer. The experimental results also show that the calculation time for a single prediction of the proposed model is 0.000247s. It indicates that the algorithm has low complexity and meets the real-time requirements of 6G mmWave UAV.

## 5. Conclusions

In this paper, a Transformer-based air-to-ground mmWave predictive model for 6G UAV communications has been proposed for the first time by integrating the advantages of CNN and the self-attention mechanism. Based on the measurement validated WI software, mmWave UAV A2G channel datasets with multiple frequency bands (10 GHz, 28 GHz, and 38 GHz), multiple altitudes (40 m, 70 m, and 100 m), and multiple locations have been constructed. The typical mmWave UAV channel characteristics have been systematically analyzed, such as the received power, KF, RMS DS, and RMS AS. In addition, Transformer-based predictive channel modeling has been carried out based on the constructed 6G mmWave UAV channel datasets. The proposed model has selected Tx and Rx coordinates, LoS distances, Tx and Rx altitude differences, frequency bands, and location tags as the network input, and received power, KF, RMS DS, and RMS AS as the network output. The results show that the Transformer-based model exhibits excellent prediction performance on channel characteristics such as received power, KF, RMS DS and RMS AS. Furthermore, the Transformer-based model shows significant robustness in complex situations and significantly outperforms CNN, BP-NN, and Attention-BiLSTM models. In conclusion, the Transformer-based model has enhanced the understanding of UAV A2G communication channel data in the mmWave band using CNN and self-attention mechanisms to improve prediction accuracy. This approach aimed to provide a novel prediction method in mmWave UAV communication channel modeling. In future work, we plan to further optimize the network and integrate more sensory information to enhance the network’s understanding of information from more complex environments.

## Figures and Tables

**Table 1:** The details of material parameters.

Material	Permittivity	Conductivity (S/m)	Thickness (m)
Wet earth	25	0.02	0
Sea water	81	20	0
Concrete	7	0.015	0.3

**Table 2:** The details of simulation parameters.

Scenario	Flight Position 1	Flight Position 2
Frequency	10 GHz/28 GHz/38 GHz	10 GHz/28 GHz/38 GHz
Bandwidth	500 MHz	500 MHz
Transmit Power	10 dBm	10 dBm
Flight Altitude	40 m/70 m/100 m	40 m/70 m/100 m
Flight Trajectory	Hover	Hover
Vehicle Travel Distance	199 m	199 m
Travel Speed	5 m/s	5 m/s
Antenna Pattern	2 × 2 antenna array	2 × 2 antenna array
Reflection/Diffraction/Transmission	6/1/0	6/1/0

**Table 3:** The details of the proposed model.

Parameter	Value
Conv Kernel Size	4 × 4
Dimension of Conv	64
Dropout	0.01
Dimension of FC1	input: 64, output: 11
Dimension of FC2	input: 11, output: 33
Dimension of FC3	input: 33, output: 11
Dimension of FC4	input: 11, output: 64
Dimension of FC5	input: 64, output: 7
Dimension of Self-Attention Head	6
Dimension of Q, K, and V	32

**Table 4:** Comparison of quantitative results of RMS DS at UAV flight position 1 for different communication frequencies and different flight altitudes.

Frequency	Flight Altitude	RMSE (ns)	MAPE	MAE (ns)
10 GHz	40 m	7.231	0.032	4.986
	70 m	5.841	0.028	4.167
	100 m	8.449	0.084	5.745
28 GHz	40 m	8.871	0.035	4.938
	70 m	4.075	0.020	2.615
	100 m	10.190	0.102	6.711
38 GHz	40 m	5.806	0.058	2.615
	70 m	1.345	0.016	0.924
	100 m	6.638	0.193	4.127

**Table 5:** Comparison of quantitative results of RMS DS at UAV flight position 2 for different communication frequencies and different flight altitudes.

Frequency	Flight Altitude	RMSE (ns)	MAPE	MAE (ns)
10 GHz	40 m	3.117	0.015	2.212
	70 m	2.098	0.006	1.046
	100 m	1.648	0.006	0.938
28 GHz	40 m	2.950	0.015	2.170
	70 m	1.855	0.006	0.989
	100 m	0.969	0.004	0.673
38 GHz	40 m	1.339	0.018	1.006
	70 m	0.470	0.004	0.302
	100 m	0.429	0.004	0.311

**Table 6:** Comparison of quantitative results of KF at UAV flight position 1 for different communication frequencies and different flight altitudes.

Frequency	Flight Altitude	RMSE (dB)	MAPE	MAE (dB)
10 GHz	40 m	1.458	0.025	0.996
	70 m	0.125	0.002	0.087
	100 m	0.088	0.002	0.066
28 GHz	40 m	2.529	0.033	1.371
	70 m	0.117	0.002	0.075
	100 m	0.082	0.002	0.055
38 GHz	40 m	2.113	0.032	1.351
	70 m	0.105	0.002	0.069
	100 m	0.076	0.002	0.060

**Table 7:** Comparison of quantitative results of KF at UAV flight position 2 for different communication frequencies and different flight altitudes.

Frequency	Flight Altitude	RMSE (dB)	MAPE	MAE (dB)
10 GHz	40 m	0.063	0.0012	0.044
	70 m	0.013	0.0003	0.011
	100 m	0.011	0.0002	0.008
28 GHz	40 m	0.064	0.0013	0.049
	70 m	0.021	0.0005	0.017
	100 m	0.010	0.0002	0.008
38 GHz	40 m	0.065	0.0014	0.051
	70 m	0.017	0.0004	0.014
	100 m	0.013	0.0003	0.010

**Table 8:** Comparison of quantitative results of received power at UAV flight position 1 for different communication frequencies and different flight altitudes.

Frequency	Flight Altitude	RMSE (dBm)	MAPE	MAE (dBm)
10 GHz	40 m	0.316	0.003	0.262
	70 m	0.092	0.001	0.071
	100 m	0.130	0.001	0.095
28 GHz	40 m	0.351	0.002	0.227
	70 m	0.099	0.001	0.076
	100 m	0.094	0.001	0.076
38 GHz	40 m	0.287	0.002	0.199
	70 m	0.068	0.001	0.054
	100 m	0.086	0.001	0.063

**Table 9:** Comparison of quantitative results of received power at UAV flight position 2 for different communication frequencies and different flight altitudes.

Frequency	Flight Altitude	RMSE (dBm)	MAPE	MAE (dBm)
10 GHz	40 m	0.087	0.0009	0.072
	70 m	0.027	0.0003	0.022
	100 m	0.024	0.0002	0.018
28 GHz	40 m	0.107	0.0009	0.082
	70 m	0.036	0.0003	0.027
	100 m	0.026	0.0002	0.020
38 GHz	40 m	0.095	0.0009	0.077
	70 m	0.032	0.0003	0.023
	100 m	0.023	0.0002	0.019

**Table 10:** Comparison of quantitative results of RMS AAOA at UAV flight position 1 for different communication frequencies and different flight altitudes.

Frequency	Flight Altitude	RMSE (°)	MAPE	MAE (°)
10 GHz	40 m	5.942	0.931	4.227
	70 m	1.919	0.634	1.371
	100 m	2.126	0.493	1.237
28 GHz	40 m	5.808	1.197	4.257
	70 m	1.282	0.478	0.864
	100 m	2.496	0.535	1.397
38 GHz	40 m	5.028	1.133	3.526
	70 m	0.404	0.405	0.290
	100 m	1.342	0.547	0.720

**Table 11:** Comparison of quantitative results of RMS AAOA at UAV flight position 2 for different communication frequencies and different flight altitudes.

Frequency	Flight Altitude	RMSE (°)	MAPE	MAE (°)
10 GHz	40 m	0.591	0.081	0.416
	70 m	0.133	0.016	0.102
	100 m	0.147	0.011	0.115
28 GHz	40 m	0.513	0.055	0.373
	70 m	0.198	0.023	0.141
	100 m	0.208	0.014	0.165
38 GHz	40 m	0.313	0.092	0.206
	70 m	0.059	0.018	0.049
	100 m	0.088	0.019	0.074

**Table 12:** Comparison of quantitative results of RMS EAOA at UAV flight position 1 for different communication frequencies and different flight altitudes.

Frequency	Flight Altitude	RMSE (°)	MAPE	MAE (°)
10 GHz	40 m	0.900	0.104	0.685
	70 m	0.814	0.026	0.633
	100 m	0.538	0.013	0.427
28 GHz	40 m	0.760	0.231	0.469
	70 m	0.505	0.023	0.354
	100 m	0.376	0.010	0.306
38 GHz	40 m	0.625	0.188	0.511
	70 m	0.542	0.016	0.310
	100 m	0.512	0.013	0.398

**Table 13:** Comparison of quantitative results of RMS EAOA at UAV flight position 2 for different communication frequencies and different flight altitudes.

Frequency	Flight Altitude	RMSE (°)	MAPE	MAE (°)
10 GHz	40 m	0.483	0.017	0.368
	70 m	0.222	0.004	0.174
	100 m	0.260	0.004	0.205
28 GHz	40 m	0.472	0.013	0.327
	70 m	0.249	0.006	0.201
	100 m	0.221	0.004	0.177
38 GHz	40 m	0.543	0.013	0.353
	70 m	0.182	0.003	0.138
	100 m	0.287	0.004	0.195

**Table 14:** Comparison of quantitative results of RMS AAOD at UAV flight position 1 for different communication frequencies and different flight altitudes.

Frequency	Flight Altitude	RMSE (°)	MAPE	MAE (°)
10 GHz	40 m	2.044	0.035	1.156
	70 m	0.782	0.021	0.570
	100 m	0.818	0.085	0.605
28 GHz	40 m	1.699	0.028	0.844
	70 m	0.601	0.016	0.416
	100 m	0.921	0.101	0.642
38 GHz	40 m	0.828	0.043	0.533
	70 m	0.245	0.014	0.153
	100 m	0.530	0.155	0.387

**Table 15:** Comparison of quantitative results of RMS AAOD at UAV flight position 2 for different communication frequencies and different flight altitudes.

Frequency	Flight Altitude	RMSE (°)	MAPE	MAE (°)
10 GHz	40 m	0.206	0.087	0.150
	70 m	0.109	0.035	0.068
	100 m	0.113	0.024	0.072
28 GHz	40 m	0.255	0.095	0.158
	70 m	0.109	0.051	0.084
	100 m	0.087	0.021	0.057
38 GHz	40 m	0.189	0.133	0.113
	70 m	0.034	0.028	0.024
	100 m	0.039	0.029	0.027

**Table 16:** Comparison of quantitative results of RMS EAOD at UAV flight position 1 for different communication frequencies and different flight altitudes.

Frequency	Flight Altitude	RMSE (°)	MAPE	MAE (°)
10 GHz	40 m	0.083	0.039	0.067
	70 m	0.106	0.026	0.080
	100 m	0.197	0.220	0.156
28 GHz	40 m	0.142	0.082	0.088
	70 m	0.081	0.020	0.056
	100 m	0.228	0.133	0.141
38 GHz	40 m	0.102	0.097	0.073
	70 m	0.028	0.017	0.021
	100 m	0.140	0.073	0.095

**Table 17:** Comparison of quantitative results of RMS EAOD at UAV flight position 2 for different communication frequencies and different flight altitudes.

Frequency	Flight Altitude	RMSE (°)	MAPE	MAE (°)
10 GHz	40 m	0.054	0.012	0.031
	70 m	0.030	0.004	0.017
	100 m	0.063	0.006	0.038
28 GHz	40 m	0.044	0.012	0.029
	70 m	0.023	0.003	0.013
	100 m	0.033	0.004	0.022
38 GHz	40 m	0.041	0.021	0.022
	70 m	0.008	0.003	0.005
	100 m	0.017	0.004	0.012

**Table 18:** Comparison of prediction performance in characteristics prediction for different models using merged datasets.

Channel Characteristic	Network	RMSE	MAPE	MAE
Received power (dBm)	Proposed model	0.172	0.001	0.118
	BP-NN	0.497	0.004	0.381
	CNN	0.250	0.002	0.158
	Attention-BiLSTM	0.448	0.004	0.331
RMS DS (ns)	Proposed model	4.577	0.050	2.769
	BP-NN	26.112	0.797	11.267
	CNN	5.961	0.169	3.370
	Attention-BiLSTM	8.347	0.205	5.713
KF (dB)	Proposed model	0.745	0.006	0.226
	BP-NN	1.675	0.020	0.776
	CNN	1.538	0.012	0.502
	Attention-BiLSTM	1.599	0.014	0.574
RMS AAOA (°)	Proposed model	1.549	0.229	0.708
	BP-NN	2.666	1.047	1.882
	CNN	2.811	0.505	1.198
	Attention-BiLSTM	2.917	0.763	1.645
RMS EAOA (°)	Proposed model	0.375	0.021	0.285
	BP-NN	1.458	0.092	1.147
	CNN	0.608	0.042	0.408
	Attention-BiLSTM	0.713	0.056	0.560
RMS AAOD (°)	Proposed model	0.864	0.096	0.450
	BP-NN	5.432	0.941	2.265
	CNN	1.094	0.156	0.574
	Attention-BiLSTM	1.415	0.344	0.901
RMS EAOD (°)	Proposed model	0.074	0.039	0.053
	BP-NN	0.496	0.749	0.317
	CNN	0.123	0.116	0.082
	Attention-BiLSTM	0.160	0.177	0.121

## Data Availability

Data will be available upon request to the corresponding author.

## Abbreviations

The following abbreviations are used in this paper:

6G	6th-generation
UAV	Unmanned Aerial Vehicle
A2G	Air to Ground
DL	Deep Learning
ANN	Artificial Neural Network
BP-NN	Backpropagation Neural Network
CNN	Convolutional Neural Network
GBSM	Geometry-Based Stochastic Model
KF	Rician K-Factor
LoS	Line of Sight
NLoS	Non-Line of Sight
LSTM	Long Short-Term Memory
Attention-BiLSTM	Attention-based Bidirectional Long Short-Term Memory
MAE	Mean Absolute Error
MAPE	Mean Absolute Percentage Error
RMSE	Root Mean Squared Error
MIMO	Multiple-Input Multiple-Output
mmWave	Millimeter Wave
MPCs	Multipath Components
PL	Path Loss
RMS AAOA	Root Mean Squared Azimuth Angle of Arrival
RMS AAOD	Root Mean Squared Azimuth Angle of Departure
RMS EAOA	Root Mean Squared Elevation Angle of Arrival
RMS EAOD	Root Mean Squared Elevation Angle of Departure
RMS AS	Root Mean Squared Angle Spread
RMS DS	Root Mean Square Delay Spread
RT	Ray-Tracing
Rx	Receiver
Tx	Transmitter
WI	Wireless InSite
